# Historical review of the Indonesian government's response to health crisis: From the Spanish flu to the COVID-19 pandemic

**DOI:** 10.1016/j.onehlt.2025.101067

**Published:** 2025-05-10

**Authors:** Ahmad Zaini Miftah, Ida Widianingsih, Connie Hoe, Irvan Afriandi

**Affiliations:** a Public Administration Department, Faculty of Social and Political Sciences, Universitas Padjadjaran, Bandung, Indonesia; b Center for Decentralization and Participatory Development Research, Faculty of Social and Political Sciences, Universitas Padjadjaran, Bandung, Indonesia; c Public Finance Administration Study Program, Applied Science Department, Vocational School, Universitas Padjadjaran, Bandung, Indonesia; d International Health Department, Johns Hopkins Bloomberg School of Public Health, Baltimore, USA; e Heidelberg Institute of Global Health, Heidelberg University, Heidelberg, Germany; f Department of Public Health, Faculty of Medicine, Universitas Padjadjaran, Sumedang, Indonesia

**Keywords:** Health policy, Crisis-response, Indigenous knowledge, Plague, Zoonotic

## Abstract

In the current post-pandemic era, One Health has emerged as an important notion, integrating concern for human, animal, and environmental health in addressing the threat of communicable disease. Communicable diseases are not a new phenomenon, as evidenced by the Spanish Flu, the Asian Flu Pandemic, Swine Flu, Bird Flu, SARS, MERS, and others. However, Indonesia has not been able to respond to the Covid-19 pandemic quickly. This study employs a retrospective policy analysis approach to the government's policy responses to health crises stemming in Indonesia, spanning from the Spanish flu pandemic to the COVID-19 pandemic. The results of this study found that the implementation of One Health in the management of infectious diseases needs to reform the national health system. It requires aspects of One Health that are proportionally integrated between humans, animals, and the environment. With this historical approach, how the Dutch East Indies government and the Indonesian government handled the outbreak becomes a lesson for improving policies. Government needs to integrate policies between sectors to minimize policy barriers and rigid segmentations that hinder coordination, as well as proportional financial funds.

## Graphical abstract

**Figure:**
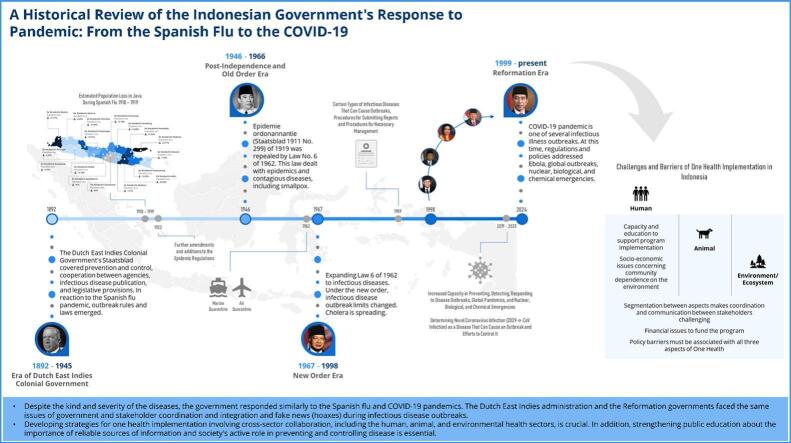

## 1 Introduction

Communicable diseases, such as the COVID-19 pandemic, influence the world's health policies. They trigger the development of new policies such as social restrictions and behavior changes, and spark debate about the government's responsibility in protecting public health and strengthening the public health system [[1], [2], [3]]. Health crises due to communicable diseases are not new occurrences; historical examples include the Spanish Flu, which was caused by the H1N1 influenza virus, the Asian Flu Pandemic, Swine Flu, Bird Flu, SARS, MERS, and others [[4], [5], [6]]. Most of these communicable diseases are zoonotic, meaning they originate in animas can be transmitted to humans [7]. Furthermore, communicable diseases persistently reemerge, underscoring the necessity for health system and policies capable of addressing the complexity of the issue and its impacts [[7], [8], [9]].

Unfortunately, as COVID-19 has demonstrated, these health crises are complex cross-sectoral issues and pose a challenge for governments around the world [[10], [11], [12], [13], [14]]. The complexity of managing a pandemic necessitates coordination, which often leads to conflicts due to differing perceptions and interests among policy actors from various sectors [[15], [16], [17], [18]], especially in health policy responses, which are technically complex, technical expertise is crucial because these responses require understanding and assessment from experts, rather than relying solely on normative considerations in policy making [2,17].

There are various policies implemented by countries in the world to deal with the spread of outbreaks, especially Covid-19, such as social distancing policies, wearing masks, self-quarantine, and others [14]. In addition, various task forces were also formed to handle pandemics, economic recovery, and public health management [14,19,20]. These policies have an impact on the lives of people whose mobility and movement are limited due to the quarantine policy due to the trade-off between the health and economic sectors [21] The economic sector is hampered by a significant decline in national income [22] With that, the government has implemented various aggressive monetary and fiscal policies to counteract this [23].

‘One World, One Health’ is an important theme emphasizing the integration of human and animal health and environmental priorities in managing the threat of communicable disease [[24], [25], [26], [27]]. This concept departs from the understanding that around 60 % of diseases experienced by humans originate from pathogens that are transmitted between animals and humans [28,29]. Calvin Schwabe, an epidemiologist, explained in 1964 that zoonic diseases could only be handled by collaboration between the fields of veterinary medicine, human health, and environmental science (Chidinma Okpua et al., 2023). Then it developed into the One Health initiative, not only collaborates the health system, but also requires social, and political roles in controlling communicable diseases [24].

The success of ‘One Health’ requires unified framing of various sectors in responding to the complexity and impact of communicable diseases such as pandemics as cross-sectoral issues [24,30,31]. The One Health approach can be implemented at multiple levels, ranging from the community to the global level, which involves shared governance, shared responsibility and accountability, communication, collaboration and the capacity required to address the associated co-benefits, risks, trade-offs and opportunities [32]. Based on research conducted by Ruckert (2020), to significantly improve the management of the outbreak and COVID-19 by focusing on the areas of integrated surveillance infrastructure, stakeholder coordination and collaboration, regulation of transmission hotspots, and fair disease management.

For centuries, strategies such as isolation, quarantine and restrictions on population movement are still believed to have helped suppress the spread of the epidemic. Early monitoring of water-borne pathogens, vector-borne diseases and zoonotic spillovers is very important to detect the risk of emerging infectious disease threats [6]. Handling communicable diseases is a challenge for the government in creating integrated policies involving various sectors [30,[33], [34], [35], [36]]. Thus, the government is encouraged to respond with innovative and integrative policies in managing health disaster emergencies and their impacts [2,37]. Even though there have been efforts made to deal with recurrent outbreaks in the past, this has not been able to prevent the negative impacts that have occurred, so a review of the government's response to previous outbreaks is needed [38].

A study like this has been carried out in Nigeria in a study conducted by Olumade et al.[38], while in Indonesia no similar literature has been found. In fact, in Indonesia, the response still faces various obstacles such as ineffective coordination between stakeholders, inadequate policies, and more focus on the economy than health [39,40]. In addition, the Indonesian government at that time was unable to respond quickly to Covid-19, taking two months to react after Covid-19 was discovered [41]. Due to this practical gap, this study has a research question: what is the government's response and what policies have been formulated to overcome communicable diseases, from the Spanish flu to the COVID-19 pandemic, with a One Health approach? This study aims to describe the government's response and what policies formulated to address cross-sectoral communicable disease issues, spanning from the outbreak of the Spanish flu to handling the COVID-19 pandemic considering the one health approach.

## 2 Material and methods

**Fig. 1:**
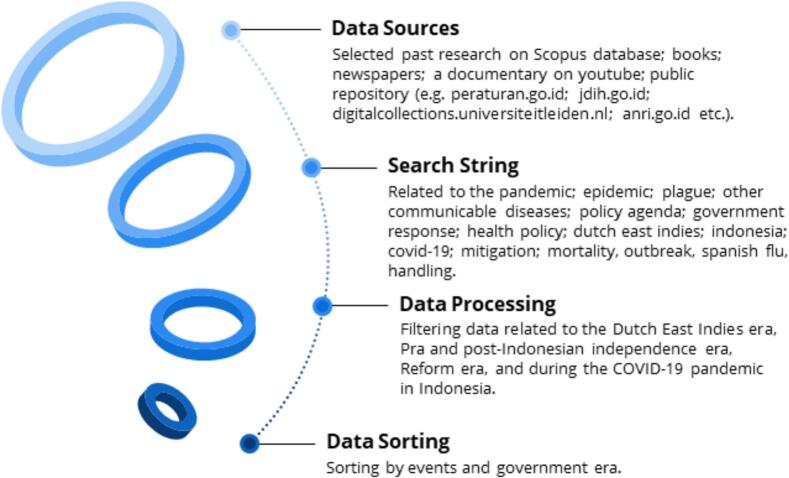
Summary of research activities.

This study employs a retrospective policy analysis approach [[42], [43], [44], [45]] to map the policy responses the government issued in dealing with the health crisis resulting from pandemics, epidemics and other disease outbreaks in Indonesia [46] since 1892–2020. In this study, the elaboration of policy response is divided into two parts, namely the pre-independence phase and the post-independence phase. The pre-independence section includes the Dutch East Indies Colonial Government phase, while the post-independence section is divided into the phases of parliamentary democracy, the old order, the new order, and the reform order up to the present. Data sources in this study were taken from articles, books, news, documentaries on YouTube, and public data repositories. The data for this study were collected from publicly accessible platforms, in accordance with LIPI (Indonesian Institute of Sciences) Regulation No. 19/2019, which exempts secondary data and publicly available information from ethical clearance requirements. Data from the University of Melbourne Forum and YouTube were utilized based on their Terms and Privacy policies, which permit the reproduction, distribution, use, and creation of derivative works from their content [47,48]. The data source described below (see Fig. 1).

The articles used in this research were searched on Google Scholar, Scopus, and manual searches on the Google search engine. In searching for relevant articles for this research, two languages were used, namely Indonesian and English, with the following keywords (see Table 1):

**Table 1:** Keywords for article searches.

Keyword	“response”;”policy”;”handling”; mitigation”;”Spanish flu”;”historic”;”outbreak”;”epidemic”;”pandemic”;”communicable disease”;”mortality”;”Indonesia”

Meanwhile, the keywords used to search for policies relevant to this research use Indonesian (INA) and Dutch (NED) as follows (see Table 2):

**Table 2:** Keywords for Policy Searches.

Keyword	Indonesian (INA)	Dutch (NED)
	“wabah”; “pandemi”; “endemi”; “penyakit menular”; “indonesia”; “penanganan”; “respon”; “pengendalian”; “kebijakan”; “penanggulangan”; “hindia belanda”; “flu spanyol”; “pes”; “kolera”; “penyakit”	“pest”; “quarantaine”; “epidemie”; “influenza”; “dutch east indies”

With many policies governing communicable diseases, we apply the following criteria:

• Inclusion criteria: discusses responses to outbreaks; published on the government's official website.
• Exclusion criteria: does not discuss aspects of preventing, handling, or controlling outbreaks; a policy document could not be found.

Afterwards, the policies found were analyzed using ATLAS.ti software. ATLAS.ti is an effective tool for organizing, recording, and interpreting qualitative data in research [49]. The data gathered is subsequently coded and mapped using the network feature of this software.

## 3 Results

### 3.1 Historical review of policies for handling communicable disease in Indonesia

**Fig. 2:**
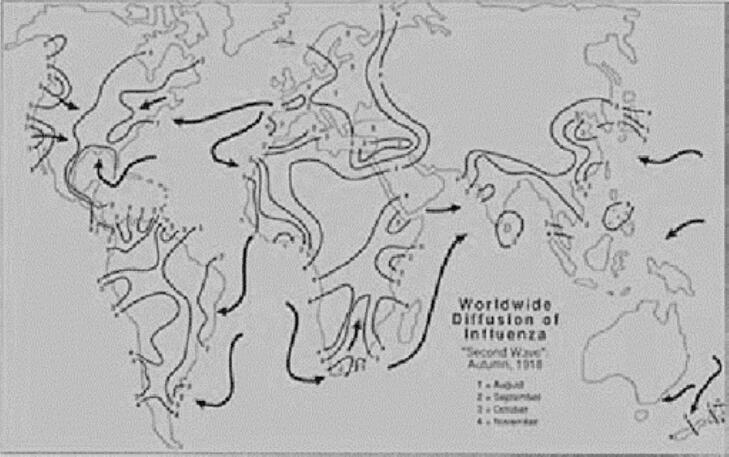
Global Spread of The Spanish Flu. Source: Patterson & Pyle, 1991.

The global spread of the Spanish Flu was immense. Fig. 2 illustrates its worldwide transmission. The virus spread from America to Europe during the fifth year of World War I, facilitated by the large-scale mobilization of civilians and military personnel [50]. The first recorded case occurred in Kansas, United States, in 1918. The pandemic lasted from March 1918 to June 1919 [51]. Its spread was intensified by the lack of medical resources, including vaccines and effective treatments, as well as heavy reliance on public health measures such as quarantine [52]. Moreover, restrictions on the dissemination of information further amplified the impact of the outbreak [53].

There were three distinct waves of transmission over a period of twelve months, occurring in spring, fall, and winter [54]. The map includes directional arrows that indicate the flow and timeline of the virus's second wave, particularly in the autumn months—August, September, October, and November of 1918. The following outlines the key phases of the global spread of the Spanish Flu [51].

• First wave: During the initial wave in 1918, the Spanish Flu began spreading across the European continent via the western coasts, affecting countries such as France, the United Kingdom, Italy, and Spain. By May, it had reached North Africa and Mumbai, India. In the following month, the first cases were reported in China, followed by southern transmission to Australia in July.
• Second wave: The second wave saw the virus spread from North to Central and South America, as well as from West Africa to both West and South Africa. By the end of September, the virus had reached nearly all of Europe. It subsequently spread to Northern Asia and re-emerged in China. The majority of deaths caused by the Spanish Flu occurred during this second wave.
• Third wave: Australia experienced its third wave between late 1918 and early 1919. This wave recorded fewer cases compared to the second. The pandemic was declared over in May 1919 in the Northern Hemisphere, while in some countries such as Japan, it persisted until 1920.

**Fig. 3:**
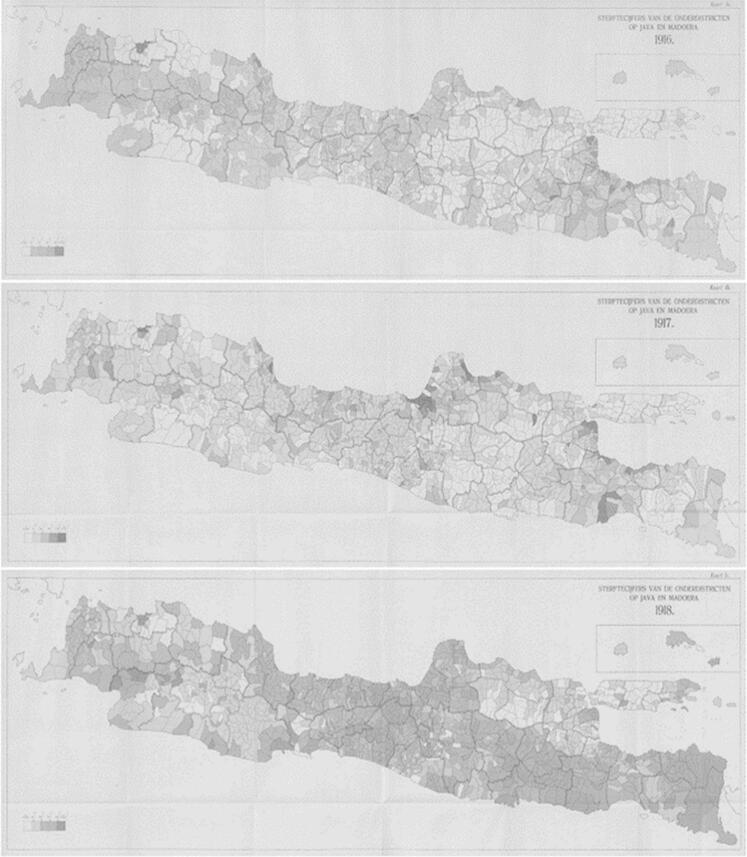
Mortality rates in the subdistricts of Java and Madura (1916–1918). Source: Uittreksel, 1922; Winckel, 1919.

Moreover, when the Spanish flu epidemic struck the Dutch East Indies region, it resulted in a significant number of causalities, as also happened in Europe (see Fig. 3) [50,52,53]. The map titled “*Sterftecijfers van de Onderafdeelingen op Java en Madoera*” translates to “Mortality rates in the administrative subdivisions (*onderafdelingen*) of Java and Madura” and presents data for three consecutive years: 1916 (top), 1917 (middle), and 1918 (bottom). The shading reflects the severity of mortality rates, with darker areas indicating higher death tolls. This document is sourced from *Uittreksel uit het jaarverslag van den Burgerlijken Geneeskundigen Dienst in Nederlandsch-Indië* over 1919 which means Summary of the annual report of the Civil Medical Service in the Dutch East Indies for the year 1919. Spanish flu also spread in Indonesia during World War I. In a previous study, it was stated that the population loss in Indonesia was around 1.5 million people. However, after further research, it was found that in Java alone there were around 4 million people [55].

**Fig. 4:**
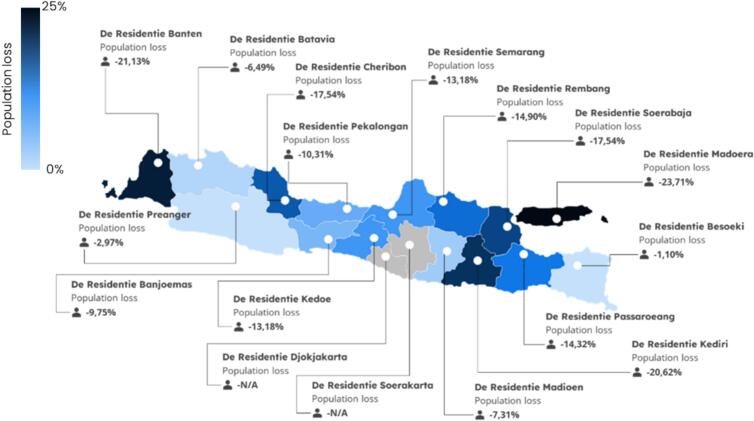
Residencies of java and estimated population loss in java during Spanish flu 1918–1919. Source: Adapted from Chandra, 2013; Lie, 2020.

From a historical perspective, the Spanish flu that afflicted Indonesia, then under the governance of the Dutch East Indies Colonial Government, resulted in a significant loss of life. The estimated death toll from the Spanish flu in Indonesia ranged from 1.5 to 4.37 million people [54,55,56]. In fact, several regions such as Madura, Banten and Kediri lost 20 % of their population due to this pandemic (Lie, 2022) (see Fig. 4). Like the government's response to the COVID-19 pandemic, the Dutch East Indies Government's response at that time was considered very slow and even considered this incident to be just a common cold and Department of Health of the Dutch East Indies *(Burgerlijken Geneeskundigen Dienst, BGD)* categorized it as part of the cholera epidemic and concluded that implementing mass vaccination would not resolve the issue [55,[57], [58], [59]]. Indeed, indications of a pandemic in Batavia at this time were detected in June 1918 [60]. However, inadequate communication and coordination between central and regional governments, coupled with the absence of a legal framework governing the management of outbreaks in the Dutch East Indies, compelled the government to take matters into its own hands. Consequently, the authorities responded by devising and implementing their own policies to address the pandemic within each residency, aiming to mitigate its impact [60].

Findings showed that Indonesia has a long history of experiencing communicable diseases such as pandemics, epidemics and other outbreaks. Starting from cross-sectoral crises that cause pressure on the health, economic and social systems, to threatening political stability due to the COVID-19 pandemic [22]. The government faces uncertainty in formulating policies, so innovative and integrative policies are needed as a response to facing the health crisis [2,30,37]. Responses in dealing with cross-sectoral crisis issues such as pandemics do not always come from the government, but also from communities, local wisdom, and lessons from past history as material for formulating policy agendas [21,45,61,62]. Moreover, health policies are so complex [2,17]. Table 3 shows the communicable diseases that occurred in Indonesia from 1800 to 2023.

**Table 3:** Communicable Disease in Indonesia 1800–2023.

Location	Disease Name	Year	# Patients	# Fatalities
Java Island	Smallpox	1800		102
Java Island	Smallpox	1910		
Malang	Pest	1911		2100
Surabaya	Cholera	1912		4230
East Java	Pest	1914		15,000
Indonesia (national)/Dutch East Indies	H1N1	1917–1918		1.5–4.37 million
Surabaya	Cholera	1918		2343
Surabaya	Cholera	1919		3852
Surabaya	Cholera	1920		3
Surabaya	Cholera	1921		1
Surabaya	Cholera	1922		9759
Surakarta Residency	Pest	1924		4679
Madura	Leprosy	1930		1200
Surabaya	Tuberculosis (TBC)	1935		32
Surabaya	Tuberculosis (TBC)	1936		29
Surabaya	Tuberculosis (TBC)	1937		27
Surabaya	Tuberculosis (TBC)	1938		31
Surabaya	Tuberculosis (TBC)	1939		22
Surabaya	Tuberculosis (TBC)	1940		24
Jakarta	Smallpox	1964	2358	
Indonesia (some provinces province)	Yaws	2000	17,085	
Indonesia (national)	Malaria	2012	417,819	
Indonesia (national)	Malaria	2013	343,527	
Indonesia (national)	Malaria	2014	252,027	
Indonesia (national)	Malaria	2015	217,025	
Indonesia (national)	Malaria	2016	218,450	
Indonesia (national)	Malaria	2017	261,617	
Indonesia (national)	Malaria	2018	222,084	
Indonesia (national)	Malaria	2019	250,644	
Indonesia (national)	Malaria	2020	254,055	
Indonesia (national)	Malaria	2021	304,607	
Indonesia (national)	Malaria	2022	415,140	
Indonesia (national)	HIV/AIDS	1987–1998	<100	
Indonesia (national)	HIV/AIDS	2000	403	
Indonesia (national)	HIV/AIDS	2005–2007	2947	
Indonesia (national)	HIV/AIDS	2008	15,136	
Indonesia (national)	HIV/AIDS	2009	18,442	
Indonesia (national)	HIV/AIDS	2010	24,131	
Indonesia (national)	HIV/AIDS	2011	26,483	
Indonesia (national)	HIV/AIDS	2012	29,879	
Indonesia (national)	HIV/AIDS	2013	52,348	
Indonesia (national)	HIV/AIDS	2014	65,790	
Indonesia (national)	HIV/AIDS	2015	77,112	
Indonesia (national)	Leprosy	2015	17,202	
Indonesia (national)	Leprosy	2016	16,826	
Indonesia (national)	Leprosy	2017	15,920	
Indonesia (national)	Influenza (A) (H5N1)	2003–2009	162	
Indonesia (national)	Influenza (A) (H5N1)	2010–2014	35	
Indonesia (national)	Influenza (A) (H5N1)	2015–2019	3	
Indonesia (national)	SARS-CoV-2	2020–2023	6,784,170	161,404

*Source:* [[71], [72], [73], [74], [75], [76], [77], [78], [79], [80]]*.*

Based on Table 1, the average data found is mostly from Java Island, Madura Island, and Indonesia nationally. Meanwhile, for other regions such as Sumatra Island, Borneo Island, Papua Island, and Sulawesi Island, specific information is not available. There are 11 diseases with recorded numbers of sufferers and fatalities between 1800 and 2023.

Apart from facing the Spanish flu, in 1892, the Dutch East Indies government also faced a cholera outbreak, to which the government responded by formulating policies to deal with the fifth cholera pandemic, which occurred from 1881 to 1896. The rapid spread of cholera was also influenced by the mobility of pilgrims to and from the Mediterranean region [63]. Global conditions at that time also influenced the response and policies of the Dutch East Indies Government. Meanwhile, to deal with the Spanish flu at that time, the Dutch East Indies Colonial government formed a task force (“Influenza Commission”) to deal with the Spanish flu, tasked with handling outbreaks, finding a cure, and carrying out investigations related to the spread of the virus (cause, source of transmission, affected areas) [64].

**Fig. 5:**
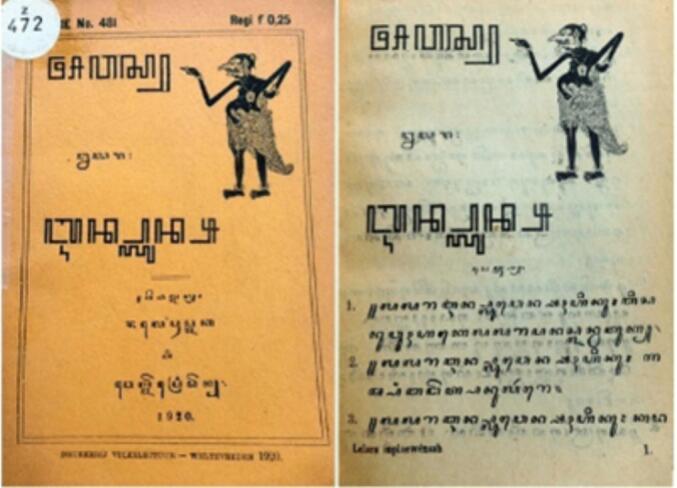
Influenza Prevention and Management Guidebook entitled “Lelara Influenza” or “Beware! Flu Disease” in Javanese Letter, published by the Balai Pustaka in 1920. Source: Looking Back at the 1918 Spanish Flu Pandemic in Colonial Indonesia | Forum, n.d., 2020.

**Fig. 6:**
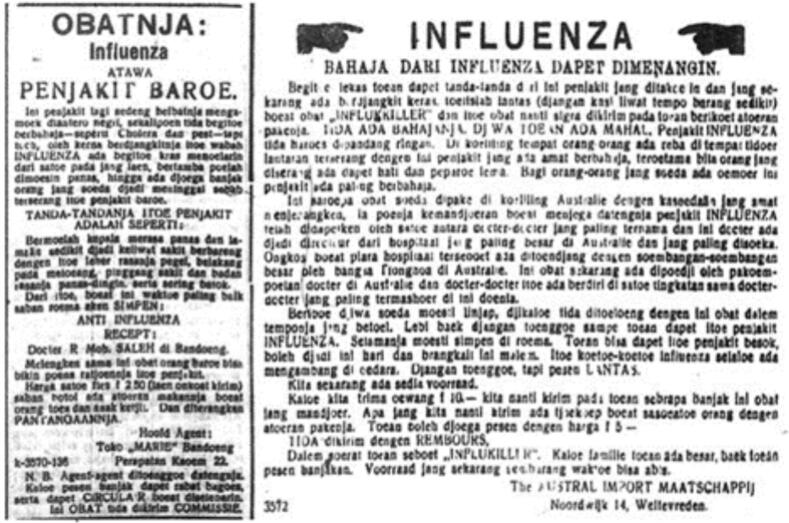
Hoax News about the Spanish Flu Published in Bataviaasch Nieuwsblad, Bromartani, and Oetoesan Hindia. Source: Lie, 2020.

The pandemic caused by the Spanish Flu in the Dutch East Indies caused poverty and inequality to increase [[65], [66], [67], [68]]. This was also influenced by regional quarantine by the Dutch East Indies Government through *Staatsblad van Nerlandsch Indie* Number 277 of 1911 concerning Granting Authority to Government Officials to Carry Out Quarantine in Areas Affected by the Plague [69]. Policies aimed at changing people's behavior as an effective strategy in controlling the epidemic control at that time, included the implementation of the socialization of “*Lelara Influenza*” guidelines which prohibited people from visiting relatives or neighbors who were suffering from influenza in hospitals; obligated to report to the orderlies if any residents were infected with influenza or deceased; and doctors and orderlies were tasked with overseeing each barrack and monitoring developments in the condition of infected village as shown in Fig. 5 [69]. However, the quarantine policy was deemed ineffective because it received many protests from various groups such as *Koninklijke Paketvaart Maatschappij* (KPM) as it disrupted their business operations [70]. In addition, the hoaxes that were influenced by the press and BGD's misunderstanding of the handling of the Spanish Flu contributed to the government's delayed response to the Spanish Flu pandemic in the Dutch East Indies as shown in Fig. 6 [60,69,70].

In the meantime, policy responses from the Indonesian and Dutch East Indies governments might be categorized according to the stages of the Indonesian political system. The Dutch East Indies colonial period, the Old Order, the New Order, and the Reformation comprise this phase. Based on the stages of the political system, the following policies have been implemented to address communicable diseases and outbreaks in Indonesia as shown in Table 4.

**Table 4:** Legal Basis for Policies for Handling Communicable Diseases in Indonesia 1892–2020.

Era	Regulation Form	Number	Year	About
Colonial Dutch East Indies	Staatsblad	45	1892	Provisions for epidemic prevention and control in the Dutch East Indies
		329	1898	The Declaration applies to the outbreak of existing provisions for the prevention and control of epidemics in the Dutch East Indies and also the establishment of special regulations in relation to diseases in the Dutch East Indies
	Staatsblad	372	1902	Changes in provisions for preventing and controlling epidemics in the Dutch East Indies (State Gazette of 1892 No. 45)
	Staatsblad	112	1902	Changes in provisions for preventing and controlling epidemics in the Dutch East Indies (State Gazette of 1892 No. 45)
	Staatsblad	277	1911	This policy gives authority to Government Officials to carry out quarantine
	Staatsblad	299	1911	Revising provisions for epidemic prevention and control
	Staatsblad	394	1911	Supplement to the Epidemic Ordinance (1911 Gazette no. 299) with agreements mostly on buildings and ships
	Staatsblad	788	1914	Further amendments to the 1911 Pandemic Gazette Ordinance No. 299
	Staatsblad	693	1914	Extension of authority granted to Regional Heads in Article 13 of the Plague Law (State Gazette 1911 no. 299)
	Staatsblad	552	1914	Inclusion of mass typhus among infectious diseases to which the Epidemic Regulations apply (State Gazette 1911 No. 299)
	Staatsblad	13/O. E	1919	Plague and Infectious Diseases
	Staatsblad	203	1919	Application of partial epidemic ordinance against Meningitis Cerebrospinalis Epidemica
	Staatsblad	676	1920	Amendments and additions to articles 4 and 5 of the Quarantine Ordinance (Staatsblad 1911 no. 277) and article 10 of the Epidemic Ordinance (Staatsblad 1911 no. 299)
	Staatsblad	781	1921	Revision of the Plague Ordinance (1911 State Gazette No. 99) and Death Certificate Ordinance (1916 State Gazette No. 612)
	Staatsblad	188	1921	Further amendments and additions to certain provisions of the Epidemic Ordinance (1911 Official Gazette No. 299)
	Staatsblad	24	1923	Further amendments and additions to the Epidemic Regulations
	Staatsblad	7	1927	Classification of paratyphus A among infectious diseases of the Epidemic Ordinance
Old Order	Government Regulations (Peraturan Pemerintah/PP)	73	1951	Declaring the Applicability of the “Epidemie Ordonnantie” (State Gazette 1911 No. 299) Against Poliomyelitis Anterior Acuta (Children's Paralytic Disease)
	Government Regulations (PP)	53	1959	Quarantine Disease
	Government Regulations (PP)	11	1961	Quarantine Disease
	Law	1	1962	Marine quarantine
	Law	2	1962	Air Quarantine
	Law	6	1962	Plague
New Order	Law	7	1968	Plague
	Law	4	1984	Infectious Disease Outbreak
	Regulation of Minister of Health	560	1989	Certain Types of Infectious Diseases That Can Cause Outbreaks, Procedures for Submitting Reports and Procedures for Necessary Management
	Government Regulation	40	1991	Management of Infectious Disease Outbreaks
	Regulation of Minister of Health	424/MENKES/SK/IV/2003	2003	Determination of SARS as a disease that can cause an epidemic and guidelines for its management
	Presidential Decree	7	2006	National Committee for Avian Influenza Control and Preparedness for Influenza Pandemics
	Presidential Instruction	1	2007	Virus Handling and Control Avian *Influenza*
	Regulation of Minister of Health	414/MENKES/SK/IV/2007	2007	Determination of a Referral Hospital for Handling Avian Influenza
	Regulation of Minister of Health	043/MENKES/SK/I/2007	2007	Malaria Training Manual
	Regulation of Minister of Health	1501/MENKES/PER/X/2010	2010	Certain Types of Infectious Diseases That Can Cause Outbreaks and Control Efforts
Reformation	Regulation of Minister of Health	5	2013	Malaria Management Guidelines
	Regulation of Minister of Health	82	2014	Management of Infectious Diseases
	Instructions of the Minister of Transportation	7	2014	Anticipate Ebola Virus Prevention
	Instructions of the Minister of Transportation	5	2014	Implementation of Middle East Respiratory Co-Navirus (Mers-Cov) Anticipation
	Presidential Instruction	4	2019	Increased Capacity in Preventing, Detecting, Responding to Disease Outbreaks, Global Pandemics, and Nuclear, Biological, and Chemical Emergencies
	Regulation of Minister of Health	HK.01.07/MENKES/556/2019	2019	National Guidelines for Malaria Management Medical Services
	Presidential decree	82	2020	Committee for Handling COVID-19 and National Economic Recovery
	Regulation of Minister of Health	HK.01.07/MENKES/104/2020	2020	Determining Novel Coronavirus Infection (2029-n-CoV Infection) as a Disease That Can Cause an Outbreak and Efforts to Control It

**Fig. 7:**
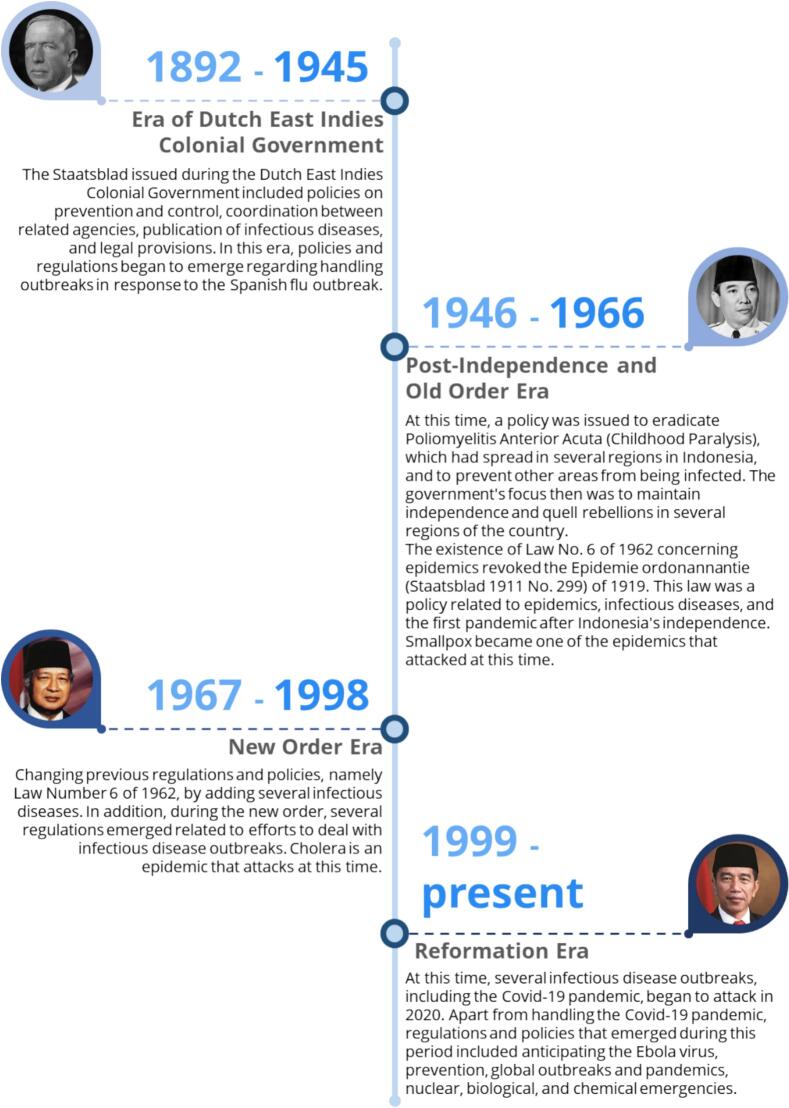
Summary of Policies in Handling Communicable Disease in Indonesia 1892–2020.

Based on the 41 policies described in the table, it shows that the outbreak is a serious matter, which can be seen from the policy responses issued by various government agencies. Parties who participate in issuing policies, such as the President, Minister of Health, Minister of State-Owned Enterprises, and the Central Government, are in accordance with their authority. By following the year timeline based on the government system era in Indonesia, can be presented in the following figure (Fig. 7).

When the Spanish flu emerged in the Dutch East Indies, the Dutch Indies Government essentially had policies in place to anticipate such outbreaks. Between 1892 and 1927, the government produced 17 policy documents that could serve as a foundation for addressing epidemics. These policies encompassed measures for preventing and controlling epidemics, authorizing regional quarantine implementation, and even regulating deaths resulting from epidemics. However, poor coordination and communication between central and regional authorities, limited understanding of the Spanish flu as a new disease, and the government's preoccupation with addressing other crises, notably the pest outbreak at the time, contributed to the delayed response in tackling the Spanish flu in the Dutch East Indies [60,70,81]. In other words, understanding the problem in the context of the policy frame is fundamental in formulating a policy agenda for handling cross-sectoral issues such as the pandemic [18,30,82,83]. Moreover, at that time, the Spanish flu also caused a trade-off due to restrictions on population movement which caused an increasing gap in economic inequality on the island of Java [[65], [66], [67]].

Then, in the early post-independence era of guided democracy (1945–1959), a policy related to epidemics, infectious diseases and pandemics was enacted. This policy was Government Regulation Number 73 of 1951 concerning Declaring the Applicability of the “*Epidemie Ordonnantie*” (State Gazette 1911 No. 299) to *Poliomyelitis Anterior Acuta* (Children's Paralytic Disease). The policy stipulates that action is necessary to eradicate the disease as it has spread to various areas in Indonesia and to prevent further infection in other regions.

Subsequently, in the Old Order era, government policy responses to communicable diseases in Indonesia through Government Regulation Number 53 of 1959 and Government Regulation Number 11 of 1961 concerning Quarantine Diseases. Then, policies in the form of laws consist of Law Number 1 of 1962 concerning Marine Quarantine, Law Number 2 of 1962 concerning Air Quarantine, and Law Number 6 of 1962 concerning Plague. Law Number 6 of 1962 concerning Outbreaks which revoked the *Epidemie Ordonnantie* (*Staatsblad* 1911 No. 299) of 1919. This law is a policy relating to epidemics, infectious diseases and the first pandemic after Indonesia's independence or 17 years since independence. This policy was established because the *Epidemie Ordonnantie* (*Staatsblad* 1911 No. 299) of 1919 was no longer relevant to the conditions of Indonesia and society. Then it was ratified by President Soekarno and promulgated on March 5, 1962 which was published in the 1962 State Gazette Number 12.

During the New Order era, which spanned from 1967 to 1998, policies regarding epidemics, infectious diseases, and pandemics were established through various legal instruments. These included laws, Minister of Health regulations, and government regulations. Among the laws enacted during this period were Law Number 7 of 1968 concerning Outbreaks and Law Number 1984 concerning Infectious Diseases. These laws provided a legal framework for addressing public health emergencies and controlling the spread of infectious diseases. Since the 1980s, vaccination of children in 204 countries in the world has increased by 50 % [84]. In addition, other policies are Minister of Health Regulation Number 560 of 1989 concerning Certain Types of Infectious Diseases that Can Cause Outbreaks, Procedures for Submitting Reports and Procedures for Necessary Management. Lastly, the policy in the form of a Government Regulation in the New Order era was Government Regulation Number 40 of 1991 concerning Management of Infectious Disease Outbreaks.

Throughout the reform era in Indonesia from 1998 to 2020, policies in the form of Presidential Regulations during the reform era in Indonesia consisted of Presidential Regulation Number 7 of 2006 concerning the National Committee for Control of Avian Influenza and Preparedness for Facing Pandemic Influenza. Then, there are two policies in the form of Presidential Instructions, namely Presidential Instruction Number 1 of 2007 concerning Handling and Control of the Avian Influenza Virus. During this time the government also began to anticipate nuclear, biological and chemical emergencies through Presidential Instruction Number 4 of 2019 concerning Increasing Capacity in Preventing, Detecting, and Responding to Disease Outbreaks, Global Pandemics, and Nuclear, Biological and Chemical Emergencies. Then, at this time, policies also emerged regarding the management of Avian Influenza, SARS, Ebola, and anticipating the Middle East Respiratory Co-Navirus (Mers-Cov).

In the history of Indonesia's outbreak policy, starting from the Dutch colonial era until before the COVID-19 pandemic, the policy included the names of diseases and pandemics. The number of diseases increased along with the issuance of these policies. The following are infectious diseases and policies that list them as shown in Table 5.

**Table 5:** List of Communicable Disease in Policy in Indonesia 1892–2020.

Disease Type	Policy
Cholera asiatica; Louse-borne typhus/Epidemic typhus	Staatsblad Number 45 of 1892, Staatsblad Number 299 of 1911, Staatsblad Number 788 of 1914, Staatsblad Number 188 of 1921, Staatsblad Number 24 of 1923, PP Number 53 of 1959, PP Number 11 of 1961, Law Number 2 of 1962, Law Number 6 of 1962, Minister of Health Regulation Number 560/MENKES/PER/VIII/1989, Minister of Health Regulation Number 1501/MENKES/PER/X/2010, Minister of Health Regulation Number 82 of 2014
Variola/Smallpox	Staatsblad Number 45 of 1892, Staatsblad Number 299 of 1911, PP Number 53 of 1959, PP Number 11 of 1961, Law Number 2 of 1962
Diphtheria	Staatsblad Number 45 of 1892, Staatsblad Number 299 of 1911, Law Number 6 of 1962, Law Number 7 of 1968, Minister of Health Regulation Number 560/MENKES/PER/VIII/1989, Minister of Health Regulation Number 1501/MENKES/PER/X/2010
Pesus/Plaque	Staatsblad Number 112 of 1902, Staatsblad Number 394 of 1911, Staatsblad Number 299 of 1911, Staatsblad Number 788 of 1914, Staatsblad Number 13/O.E of 1919, Staatsblad Number 676 of 1920, Staatsblad Number 188 of 1921, Staatsblad Number 24 of 1923, PP Number 53 of 1959, PP Number 11 of 1961, Law Number 2 of 1962, Minister of Health Regulation Number 560/MENKES/PER/VIII/1989, Minister of Health Regulation Number 1501/MENKES/PER/X/2010
Typhus/Typhoid	Staatsblad Number 552 of 1914, PP Number 53 of 1959, PP Number 11 of 1961, Law Number 2 of 1962, Law Number 7 of 1968, Minister of Health Regulation Number 560/MENKES/PER/VIII/1989
Cerebrospinal meningitis	Staatsblad Number 203 of 1919, Law Number 6 of 1962, Law Number 7 of 1968, Minister of Health Regulation Number 560/MENKES/PER/VIII/1989, Minister of Health Regulation Number 1501/MENKES/PER/X/2010, Minister of Health Regulation Number 82 of 2014
Paratyphoid	Staatsblad Nomor 7 Tahun 1927, UU Nomor 6 Tahun 1962, UU Nomor 7 Tahun 1968
Poliomyelitis anterior acuta (childhood paralysis)	Staatsblad Number 7 of 1927, Law Number 6 of 1962, Law Number 7 of 1968 PP Number 73 of 1951, Law Number 6 of 1962, Law Number 7 of 1968, Minister of Health Regulation Number 560/MENKES/PER/VIII/1989, Minister of Health Regulation Number 1501/MENKES/PER/X/2010, Minister of Health Regulation Number 82 of 2014
Febris flava/Yellow fever	PP Number 53 of 1959, PP Number 11 of 1961, Law Number 2 of 1962, Minister of Health Regulation Number 560/MENKES/PER/VIII/1989, Minister of Health Regulation Number 1501/MENKES/PER/X/2010, Minister of Health Regulation Number 82 of 2014
Febris recurrens/Relapsing Fever	PP Number 53 of 1959, PP Number 11 of 1961, Law Number 2 of 1962, Minister of Health Regulation Number 560/MENKES/PER/VIII/1989
Bacillary dysentery	Law Number 6 of 1962
Infectious liver inflammation/hepatitis	Law Number 6 of 1962 Law Number 6 of 1962, Law Number 7 of 1968, Minister of Health Regulation Number 560/MENKES/PER/VIII/1989, Minister of Health Regulation Number 1501/MENKES/PER/X/2010, Minister of Health Regulation Number 82 of 2014
Measles	Minister of Health Regulation Number 560/MENKES/PER/VIII/1989, Minister of Health Regulation Number 1501/MENKES/PER/X/2010, Minister of Health Regulation Number 82 of 2014
Rabies	Minister of Health Regulation Number 560/MENKES/PER/VIII/1989, Minister of Health Regulation Number 1501/MENKES/PER/X/2010, Minister of Health Regulation Number 82 of 2014
Influenza	Minister of Health Regulation Number 560/MENKES/PER/VIII/1989, Minister of Health Regulation Number 82 of 2014
Encephalitis	Minister of Health Regulation Number 560/MENKES/PER/VIII/1989, Minister of Health Regulation Number 82 of 2014
Dengue Hemorrhagic Fever (DBD)	Permenkes Nomor 560/MENKES/PER/VIII/1989, Surat Edaran Menteri BUMN Nomor SE-02/MBU/2004, Permenkes Nomor 1501/MENKES/PER/X/2010, Permenkes Nomor 82 Tahun 2014
Pertusis	Minister of Health Regulation Number 560/MENKES/PER/VIII/1989, Minister of Health Regulation Number 1501/MENKES/PER/X/2010, Minister of Health Regulation Number 82 of 2014
Malaria	Minister of Health Decree Number 560/MENKES/PER/VIII/1989, Minister of Health Decree Number 043/MENKES/SK/I/2007, Minister of Health Regulation Number 1501/MENKES/PER/X/2010, Minister of Health Regulation Number 5 of 2013, Minister of Health Regulation Number 82 of 2014, Minister of Health Decree Number HK.01.07/MENKES/556/2019, Minister of Health Regulation Number 82 of 2014
Anthrax	Minister of Health Regulation Number 560/MENKES/PER/VIII/1989, Minister of Health Regulation Number 1501/MENKES/PER/X/2010, Minister of Health Regulation Number 82 of 2014
Severe Acute Respiratory Syndrome (SARS)	Minister of Health Decree Number 424/MENKES/SK/IV/2003
Avian Influenza)	Presidential Decree Number 7 of 2006, Presidential Instruction Number 1 of 2007, Minister of Health Decree Number 414/MENKES/SK/IV/2007, Minister of Health Regulation Number 1501/MENKES/PER/X/2010, Minister of Health Regulation Number 82 of 2014
Influenza A (H1N1)	Minister of Health Regulation Number 1501/MENKES/PER/X/2010, Minister of Health Regulation Number 82 of 2014
Chikungunya	Minister of Health Regulation Number 1501/MENKES/PER/X/2010, Minister of Health Regulation Number 82 of 2014
Leptospirosis	Minister of Health Regulation Number 1501/MENKES/PER/X/2010, Minister of Health Regulation Number 82 of 2014
Tetanus	Minister of Health Regulation Number 82 of 2014
Rubella	Minister of Health Regulation Number 82 of 2014
Pneumococcal disease	Permenkes Nomor 82 Tahun 2014
Disease caused by Rotavirus	Permenkes Nomor 82 Tahun 2014
Disease caused by Human Papiloma Virus (HPV)	Permenkes Nomor 82 Tahun 2014
Ebola virus	Minister of Health Regulation Number 82 of 2014, Minister of Transportation Instruction Number 7 of 2014
MERS-CoV	Minister of Health Regulation Number 82 of 2014, Minister of Transportation Instruction Number 7 of 2014
Gastrointestinal infections	Minister of Health Regulation Number 82 of 2014
Sexually transmitted infections	Minister of Health Regulation Number 82 of 2014
Human Immunodeficiency Virus (HIV) infection	Minister of Health Regulation Number 82 of 2014
Respiratory tract infections	Minister of Health Regulation Number 82 of 2014
Leprosy	Minister of Health Regulation Number 82 of 2014
Yaws	Minister of Health Regulation Number 82 of 2014
Filaris and worms	Minister of Health Regulation Number 82 of 2014
Schistosomiasis	Minister of Health Regulation Number 82 of 2014
Toxoplasma	Minister of Health Regulation Number 82 of 2014
West Nile	Minister of Health Regulation Number 82 of 2014

Based on this table, there are 42 infectious diseases that have been identified in Indonesia from the Dutch East Indies Colonial period to the Reformation era. The list of infectious diseases is summarized in certain policies so that, from year to year, it continues to grow. For example, in Minister of Health Regulation Number 82 of 2014 concerning Management of Infectious Diseases, which summarizes 37 infectious diseases in Indonesia up to the year the policy was established.

**Fig. 8:**
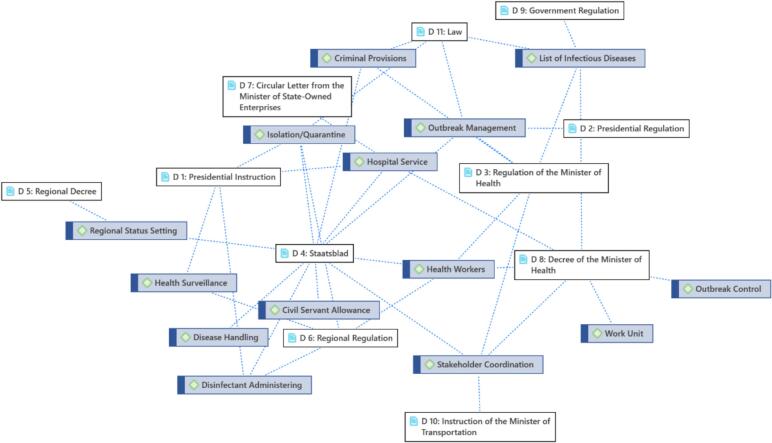
Historical context and policy response for handling communicable diseases in Indonesia in 1892–2020.

Regulations during the outbreak that occurred in Indonesia were issued by various agencies and levels of government according to their authority. With that, there are various types of policies that can be grouped as Staatsblad, Laws, Government Regulations, Ministerial Regulations, Ministerial Circulars, Ministerial Decrees, Ministerial Instructions, and other regulations at the regional level. By using ATLAS.ti software, the context of the policies issued based on the type of policy can be described as follows in Fig. 8.

The *Staatsblad* issued during the Dutch colonial era included prevention and response policies for infectious diseases, coordination between relevant stakeholders, publication of infectious diseases, and punishment provisions. Prevention efforts against infectious diseases involved medical services provision, health monitoring for sufferers, supplying disinfectants, and implementing isolation or quarantine measures. Infectious disease publications included information regarding the list of infectious diseases and the determination of a location's status as an outbreak area. Apart from that, the *Staatsblad Wijziging Van De Bepalingen Tervoorkoming En Beteugeling Bvan Epidemien In Nederlandsch-Indie* (Staatsblad 1892 No. 45) or in Indonesian ‘Changes to the provisions for preventing and controlling epidemics in the Dutch East Indies (State Gazette of 1892 No. 45) regulate the wage system for regional European employees. Wages were determined based on the distance traveled and the mode of transportation used to reach the workplace.

In addition to Laws, Government Regulations, Presidential Regulations, Ministerial Regulations, Presidential Instructions, Ministerial Instructions, Ministerial Regulations, up to Circular Letters, regulations regarding hospital services, treatment, health workers, up to outbreak handling work unit were regulated. For example, the work unit mentioned is the establishment of the National Committee for Control of Avian Influenza and Preparedness for Facing Pandemic Influenza or Komnas FBPI (Control of Bird Flu and Preparedness for Facing Pandemic Influenza) through Presidential Regulation Number 7 of 2006.

### 3.2 Handling the COVID-19 pandemic

The COVID-19 pandemic, which is an acute shock [85], tended to spread more quickly in urban areas with high mobility [61,86,87]. In Indonesia, COVID-19 has officially become a national health emergency problem through Presidential Decree Number 11 of 2020. Followed by the non-natural disaster of the spread of COVID-19 as a national disaster with Presidential Decree Number 12 of 2020 and the Large-Scale Social Restrictions policy (PSBB, *Pembatasan Sosial Berkala Besar*) in several areas where there are cases of positive COVID-19 patients as regulated in Government Regulation Number 21 of 2020.

In the initial phase of the COVID-19 pandemic in Indonesia, the response from both the central and regional governments varied and was perceived to be relatively slow. This discrepancy can be attributed to several factors, including political competition and the influence of media discourse within the public sphere. These factors may have contributed to delays in implementing cohesive and effective measures to address the pandemic at both the national and local levels [88]. The Indonesian Government's response to the health emergency resulting from the COVID-19 pandemic commenced with Decree of the President of the Republic of Indonesia Number 12 of 2020 concerning the Designation of a Non-Natural Disaster, the Spread of Corona Virus Disease 2019 (COVID-19) as a National Disaster, which was followed by the formation of a Task Force for the Acceleration of Handling COVID-19 and also Decree of Minister of Health No: HK.01.07/MENKES/104/2020 [20,89].

The COVID-19 pandemic, which had several variants, also gave rise to a response from the community based on local wisdom [62]. Both the central and regional governments sought to resolve the health crisis resulting from the COVID-19 pandemic, not only in the prevention aspect through efforts to suppress virus transmission. However, we also anticipated long-term impacts on both social and economic aspects which were emphasized by President Joko Widodo by stating that the initial foundation for economic recovery is controlling the spread of the COVID-19 virus [89].

Basically, the responses to handling the pandemic due to the Spanish flu and the COVID-19 pandemic were similar. Starting from the development of a special unit that handles pandemics, socio-economic impacts, health system governance, to the emergence of hoaxes in society. Apart from that, one thing that needs to be underlined is the importance of shared understanding in determining and implementing policies to make them more effective and efficient (policy frame in the agenda setting process) [18,31,83,[90], [91], [92]]*.* Moreover, health policies such as handling a pandemic are very complex [2,17].

In Indonesia, the COVID-19 pandemic has revealed deficiencies in the national healthcare system, particularly in its capacity to prevent and manage surges in cases and its limited healthcare resources. Important lessons learned from the pandemic include the need for better utilization of information technology, enhancing prevention and mitigation capabilities, improving risk communication, expanding healthcare facilities to handle increased caseloads, enhancing mechanisms for mobilizing healthcare financing, and promoting public adherence to health and vaccination protocols, all of which require improvement [93]. The urgency to strengthen disease prevention and the national health system underscores the necessity for comprehensive reform in Indonesia's healthcare system, enabling it to effectively confront the threats of epidemics, outbreaks, and other health emergencies.

## 4 Discussion

The response to handling communicable diseases from the Spanish flu to the COVID-19 pandemic has had the same pattern. Starting from the formation of special work units to handle outbreaks such as the influenza commission and the Task Force for Handling the COVID-19 Pandemic and National Economic Recovery, to the emergence of hoaxes and coordination problems between the central and regional governments. In other words, understanding problems in the context of a policy frame is an important process in developing integrative policy strategies in handling complex and cross-sectoral health crises [18,30,82,83].

The series of communicable diseases, from the Spanish flu to the COVID-19 pandemic, has brought awareness to the importance of integrated health system capacity to reduce the risks and impacts caused by infectious diseases [30,94,95]. Although developing the capacity of an integrated health system to face health crises is quite difficult [96], especially in developing countries where cross-sectoral collaboration is influenced by political perceptions, backgrounds and interests of actors, up to administrative barriers [2,30,97].

As mandated by the International Health Regulations (IHR), every member country must possess core capabilities to prevent, detect, and rapidly respond to public health threats. However, the findings of the Joint External Evaluation (JEE) assessment conducted in 2017 revealed deficiencies in Indonesia's technical capacity, including inadequacies in response mechanisms for potential infectious and zoonotic diseases, integration and analysis of surveillance data, identification of public health risks, as well as operational readiness and response during emergency situations [98].

The One Health initiative extends beyond the healthcare sector, necessitating involvement from social and political dimensions [24]. Success in implementing the One Health approach relies on an integrated framework that spans various sectors. Given the complexity and impact of communicable diseases, cross-sectoral understanding and response are crucial. Recognizing the interconnectedness between human, animal, and environmental health, and engaging multiple sectors and dimensions in disease control efforts, can enhance prevention and control strategies in a more effective and holistic manner [99]. Findings from this study reveal that One Health can serve as a collaborative approach to anticipate zoonotic diseases that lead to communicable diseases in Indonesia.

Key findings from JEE 2017 highlight that cross-sector coordination is a weak point in preventing, detecting and responding to public health emergencies [98]. One Health approach can be effective if there is a communication framework that supports it, and has optimal benefits when used to find innovative, impactful, sustainable and proactive solutions to disease and health maintenance [100]. In its implementation, One Health can be linked to the national health system reform framework which is currently being implemented by the Indonesian government. Strengthening the national health system is one of the strategies in the 2020–2024 Medium Term Development Plan (RPJMN, *Rencana Pembangunan Jangka Menengah Nasional*) to overcome health development problems.

With the One Health approach, experts in both animal and human health can swiftly identify and anticipate communicable diseases originating from animals. Historical research conducted in this study provides initial insights into the occurrence of various communicable diseases in Indonesia, enabling prompt preventive measures to curb disease transmission to humans. Collaboration among human and animal health experts within the One Health framework facilitates early detection and response to outbreaks of communicable diseases, leading to more effective interventions and minimizing adverse impacts. Furthermore, as zoonoses are often linked to environmental changes, the One Health approach fosters a deeper understanding of how human activities impact ecosystems and subsequently influence human and animal health. Therefore, environmental protection efforts are crucial within the One Health framework for preventing disease outbreaks. Lastly, promoting unity in policymaking is vital. The One Health approach emphasizes integration to facilitate collaboration among diverse stakeholders, including government bodies, health professionals, scientists, and communities, in developing and implementing comprehensive policies to address zoonoses. There are several challenges facing Indonesia that could hinder the implementation of the One Health approach in Indonesia, as shown in Table 6 [101,102].

**Table 6:** Challenges and Barriers of One Health Implementation in Indonesia.

	Challenges and Barriers				
Human	Capacity and education to support program implementation	Socio-economic issues concerning community dependence on the environment	Segmentation between aspects makes coordination and communication between stakeholders challenging	Financial issues to fund the program	Policy barriers must be associated with all three aspects of One Health.
Animal					
Environment/ ecosystem					

This is in line with the previous study conducted by Thursina et al. [103] with a similar topic to this study, which showed that the One Health approach addresses the complex zoonotic problems also in Indonesia. On the other hand, many health workers do not internalize and apply this approach. This is a gap. This gap causes the handling of zoonotic diseases to be fragmented and not integrated. Thursina et al. recommended that the socialization of One Health holistically needs to be a priority to manage zoonoses in Indonesia. priority to manage zoonoses in Indonesia. Meanwhile, Li et al. [104] also emphasized that in the last few decades there have been at least 55 diseases from 59 pathogens detected in China. These researchers explained that effective monitoring of the risk of zoonotic disease emergence needs to be carried out in the animal value chain, especially in wild animal hosts. Especially in wild animal hosts supported by cross-sector stakeholder collaboration.

Kementrian PPN & Bappenas (2022) emphasized the critical need for Health System Reform, particularly in response to the lessons learned from managing the COVID-19 pandemic. This highlights weaknesses within the national health system, particularly in terms of health security and resilience, and its inability to address chronic health development issues effectively. The pandemic underscored that the existing National Health System has not operated optimally, lacking clear guidance in pandemic situations. The imperative for implementing national health system reform underscores Indonesia's commitment to ensuring robust health resilience in the face of communicable disease threats, reflecting a proactive stance towards safeguarding public.

An essential aspect of reforming the national health system involves prioritizing a thorough learning process based on the experiences gained during the COVID-19 pandemic. The reform of the national health system encompasses three key stages: conducting activities, establishing frameworks or assumptions, and implementing a learning system [93], where health system learning involves three interconnected aspects, including information, deliberation, and action. National Health System reform needs to review the framework and risk factors and involve sectors in Indonesia [93]. The Ministry of National Development Planning/Bappenas is carrying out a strategy in reforming the National Health System /based on a systems and evidence-based approach, with a focus on targets based on impact, effective delivery and sustainability. Apart from that, this effort also aims to change the direction of development policies in all sectors to support health (health in all policies). The learning process carried out has the same enthusiasm to see, identify and understand a series of government responses from the Spanish flu period to the COVID-19 pandemic which is discussed in this article.

Approaches to managing communicable diseases, from the Spanish flu to the COVID-19 pandemic, exhibit similar patterns despite variations in disease type and severity. Observable patterns include the establishment of special task forces, such as the influenza commission and the Task Force for Handling the COVID-19 Pandemic and National Economic Recovery, becoming routine government responses to outbreaks and pandemics. These dedicated teams coordinate management, monitoring, and recovery efforts. Additionally, the proliferation of fake news (hoaxes) during infectious disease outbreaks, such as the COVID-19 pandemic, poses a significant challenge, potentially confusing the public and hindering efficient response efforts. Therefore, it is crucial to educate the public early on about reliable sources of information and counter the spread of misinformation. Furthermore, coordinating infectious disease control efforts between central and regional governments and other stakeholders proves challenging. This coordination issue can impede swift and effective outbreak or pandemic responses. Strengthening coordination and communication protocols across various levels of government is essential to address this challenge effectively.

Based on these three points, it is crucial to develop strategies for handling communicable diseases that involve cross-sector collaboration, including the human, animal and environmental health sectors. In addition, it is important to strengthen public education about the importance of reliable sources of information and the active role of society in preventing and controlling disease. Good coordination between central and regional governments is also a fundamental factor as an effective response to an outbreak or pandemic. However, is this sufficient? What about communicable diseases, ranging from the Spanish flu to the COVID-19 pandemic, which consistently provoke panic and uncertainty? Future research could further investigate these areas.

The ongoing National Health System reform is structured around eight strategic areas, aimed at catalyzing future health development, with Primary Health Care (PHC) emerging as the central focus within these domains. These eight strategic areas encompass the following: education and deployment of healthcare personnel, enhancement of community health centers, enhancement of hospital quality and healthcare services accessibility in remote regions, border areas, and islands, self-sufficiency in pharmaceuticals and medical equipment, health security, disease control, and immunization, healthcare financing, and the integration of information technology alongside community empowerment initiatives. If this article promotes One Health as a solution for addressing communicable diseases, then based on the eight focal areas of the reform, the authors observe that the full implementation of the One Health concept is yet to be achieved in Indonesia's national health system overhaul. These eight areas currently do not fully integrate a harmonious socioecological balance encompassing human-animal-environment interactions. Through the One Health Approach, there is a pressing need for government initiatives to underscore the significance of comprehending the impact of human activities on ecosystems and its potential repercussions on human and animal health. Consequently, prioritizing environmental protection efforts becomes pivotal within the framework of One Health.

## 5 Conclusion

This study illustrates how the One Health approach can be applied to integrate policies in reforming Indonesia's national health system. By adopting the One Health approach, the health system can enhance its effectiveness in preventing, detecting, and responding to public health risks involving human, animal, and environmental factors. Historical insights play a crucial role in shaping the future of health, particularly in addressing communicable diseases. Through the One Health approach, lessons from history can be incorporated into strategies aimed at maintaining the balance of human, animal, and environmental ecosystems. It is imperative for the national health system to not only focus on disease control and treatment but also prioritize disease prevention. Given the nature of communicable diseases, prevention programs integrating human, animal, and environmental health sectors are of utmost importance. This study recommends that the design of national health system reform in Indonesia should incorporate aspects of human, animal, and environmental health to effectively prevent communicable diseases. Also, proportional financial funds for each aspect of One Health.

This study is able to reveal how the Dutch East Indies and Indonesian governments handled communicable diseases since the colonial era using a historical approach. In addition, this study not only considers the circumstances and the number of victims of the outbreak that had occurred in Indonesia but also examines, in an interdisciplinary way, how the Indonesian government responded to it. With that, the government can use this study as a reflection and analysis of the documentation of actions that have been taken so that it can be used as a lesson learned in dealing with communicable diseases in the future.

Meanwhile the limitations of this study, only official Indonesian government websites were considered as policy sources. Further study can be conducted using a wider range of policy sources, such as archives offered by other countries such as the Netherlands or Japan. This study's data sources are limited to Indonesian, Dutch, and regional languages in Indonesia. Future research can include more diverse languages, such as Japanese. The scope of this study then expands to cover places, sufferers, fatalities, and actions enacted between the Dutch colonial period and the Covid-19 pandemic. As a result, in the following study, the scope might be broadened by examining parallels with other regions that share features with Indonesia. Aside from that, future studies necessitate particular research on One Health practices addressing outbreaks that have occurred in Indonesia.

## CRediT authorship contribution statement

**Ahmad Zaini Miftah:** Writing – review & editing, Writing – original draft, Visualization, Validation, Software, Resources, Project administration, Methodology, Investigation, Formal analysis, Data curation, Conceptualization. **Ida Widianingsih:** Writing – review & editing, Validation, Supervision, Resources, Project administration, Investigation, Funding acquisition, Data curation, Conceptualization. **Connie Hoe:** Writing – review & editing, Writing – original draft, Validation, Supervision, Resources, Formal analysis, Data curation. **Irvan Afriandi:** Writing – review & editing, Validation, Methodology, Investigation, Formal analysis, Conceptualization.

## Ethical clearance

In accordance with Indonesian regulations (LIPI No. 19/2019), secondary data, literature reviews, and publicly available information are exempt from ethical clearance. This includes data obtained from websites, public statements, films, and other published works. Additionally, platforms such as the University of Melbourne Forum and YouTube explicitly permit the use of their content under their Terms and Privacy policies, including reproduction, distribution, and the creation of derivative works.

## Declaration of competing interest

The authors declare that they have no known competing financial interests or personal relationships that could have appeared to influence the work reported in this paper.

## Data Availability

Data will be made available on request.

## References

[1] Knobler S., Mahmoud A., Lemon S., Mack A., Sivitz L., Oberholtzer K. (2021).

[2] Shigayeva A., Atun R., McKee M., Coker R. (2010). Health systems, communicable diseases and integration. Health Policy Plan..

[3] Béland D. (2010). Reconsidering policy feedback: how policies affect politics. Adm. Soc..

[4] Agrawal A., Gindodiya A., Deo K., Kashikar S., Fulzele P., Khatib N. (2021). A comparative analysis of the Spanish flu 1918 and COVID-19 pandemics. Open Public Health J..

[5] Short K.R., Kedzierska K., van de Sandt C.E. (2018). Back to the future: lessons learned from the 1918 influenza pandemic. Front. Cell. Infect. Microbiol..

[6] Piret J., Boivin G. (2021). Pandemics throughout history. Front. Microbiol..

[7] Waltner-Toews D. (2017). Zoonoses, one health and complexity: wicked problems and constructive conflict. Philos. Trans. R. Soc. B.

[8] Fouda A., Moy N. (2022). Health System Shock Frameworks Between Theory, Application, and Assessment Comment on ‘The COVID-19 System Shock Framework: Capturing Health System Innovation During the COVID-19 Pandemic’. Int. J. Health Policy Manag..

[9] Tangcharoensathien V., Panichkriangkrai W., Witthyapipopsakul W., Patcharanarumol W. (2022). COVID-19 Aftermath: Direction Towards Universal Health Coverage in Low-Income Countries Comment on ‘Health Coverage and Financial Protection in Uganda: A Political Economy Perspective’. Int. J. Health Policy Manag..

[10] Han E. (2020). Lessons learnt from easing COVID-19 restrictions: an analysis of countries and regions in Asia Pacific and Europe. Lancet.

[11] Mei C. (2020). Policy style, consistency and the effectiveness of the policy mix in China’s fight against COVID-19. Polic. Soc..

[12] Chandra S., Christensen J., Likhtman S. (2020). Connectivity and seasonality: the 1918 influenza and COVID-19 pandemics in global perspective. J. Glob. Hist..

[13] Wernli D. (2022). A complexity Lens on the COVID-19 pandemic. Int. J. Health Policy Manag..

[14] Miftah A.Z., Widianingsih I., Muhtar E.A., Sutriadi R. (2024). Behind the scenes of COVID-19 response: a social network analysis of policy actors in Bandung City. Cogent. Soc. Sci..

[15] Tosun J., Lang A. (2013). Paper Prepared for Presentation at the 7th ECPR General Conference, Bordeaux, France.

[16] Peters B.G. (2018). The challenge of policy coordination. Policy Design Pract..

[17] Trein P., Maggetti M. (2019). Patterns of policy integration and administrative coordination reforms: a comparative empirical analysis. Public Adm. Rev..

[18] Candel J.J.L. (2019). The expediency of policy integration. Policy Stud.

[19] Shin Y., Kim S., Lee S.W., An K. (2020). Identifying the planning priorities for green infrastructure within urban environments using analytic hierarchy process. Sustainability.

[20] Putera P.B., Widianingsih I., Ningrum S., Suryanto S., Rianto Y. (2022). Overcoming the COVID-19 pandemic in Indonesia: a science, technology, and innovation (STI) policy perspective. Health Policy Technol..

[21] Miftah A.Z., Widianingsih I., Muhtar E.A., Sutriadi R. (2023). Mapping netizen perception on COVID-19 pandemic: a preliminary study of policy integration for pandemic response in Bandung City. KnE Social Sci..

[22] Miftah A.Z., Widianingsih I., Muhtar E.A., Sutriadi R. (2023). Reviving a City’s economic engine: the COVID-19 pandemic impact and the private sector’s engagement in Bandung City. Sustainability.

[23] Sumaryana A., Miftah A.Z., Widianingsih I., Karlina N. (2025). Turning over a new leaf: post-Covid infrastructure development planning and financing strategies in the organizational environment of Bandung City. Reg. Sci. Policy Pract..

[24] Mwacalimba K.K., Green J. (2015). One health and development priorities in resource-constrained countries: policy lessons from avian and pandemic influenza preparedness in Zambia. Health Policy Plan..

[25] FAO, UNEP, WHO, and WOAH (2022).

[26] Bonilla-Aldana D.K., Dhama K., Rodriguez-Morales A.J. (2020). Revisiting the one health approach in the context of COVID-19: a look into the ecology of this emerging disease. Adv. Anim. Vet. Sci..

[27] Chidinma Okpua N., Mohd Shariff N., Hami R., Mohd Mujar N.M. (2023). Preventing the resurgence of Covid-19 pandemic and similar epidemics: the need for one health approach. J. Health Transl. Med..

[28] Puig B., Uskola A. (2021). Understanding pandemics such as covid-19 through the lenses of the ‘one health’ approach. Sustainability.

[29] de Jong W. (2018).

[30] Maggetti M., Trein P. (2022). Policy integration, problem-solving, and the coronavirus disease crisis: lessons for policy design. Polic. Soc..

[31] Biesbroek R., Candel J.J.L. (2020). Mechanisms for policy (dis)integration: explaining food policy and climate change adaptation policy in the Netherlands. Policy. Sci..

[32] Dar O. (2022). One Health Theory of Change. https://www.who.int/publications/m/item/one-health-theory-of-change.

[33] Djalante R., Shaw R., DeWit A. (2020). Building resilience against biological hazards and pandemics: COVID-19 and its implications for the Sendai framework. Progr. Disas. Sci..

[34] Basurto A., Dawid H., Harting P., Hepp J., Kohlweyer D. (2023). How to design virus containment policies? A joint analysis of economic and epidemic dynamics under the COVID-19 pandemic. J. Econ. Interac. Coord..

[35] Saguin K., Howlett M. (2022). Enhancing policy capacity for better policy integration: achieving the sustainable development goals in a post COVID-19 world. Sustainability.

[36] Spadaro I., Pirlone F., Bruno F., Saba G., Poggio B., Bruzzone S. (2023). Stakeholder participation in planning of a sustainable and competitive tourism destination: the Genoa integrated action plan. Sustainability.

[37] Weible C.M. (2020). COVID-19 and the policy sciences: initial reactions and perspectives. Policy. Sci..

[38] Olumade T.J. (2020).

[39] Arifin S. (2024). The quality of Indonesia’s COVID-19 legislation. Theory Pract. Legis..

[40] Mietzner M. (2020). Populist anti-scientism, religious polarisation, and institutionalised corruption: how Indonesia’s democratic decline shaped its COVID-19 response. J. Curr. Southeast Asian Affairs.

[41] Hosen N., Hammado N. (2021). Covid-19 in Asia.

[42] El-Jardali F., Bou-Karroum L., Ataya N., El-Ghali H.A., Hammoud R. (2014). A retrospective health policy analysis of the development and implementation of the voluntary health insurance system in Lebanon: learning from failure. Soc. Sci. Med..

[43] Patton C.V., Sawicki D.S., Clark J.J. (2015).

[44] Putera P.B., Widianingsih I., Ningrum S., Suryanto S., Rianto Y. (2022). Science, technology and innovation (STI) ecosystems in Indonesia (1945-2021): a historical policy analysis. Hist. Sci. Technol..

[45] Brand U., Krams M., Lenikus V., Schneider E. (2022). Contours of historical-materialist policy analysis. Crit. Policy Stud..

[46] Nanda W.D., Widianingsih I., Miftah A.Z. (2023). The linkage of digital transformation and tourism development policies in Indonesia from 1879–2022: trends and implications for the future. Sustainability.

[47] University of Melbourne Online Terms and Privacy. https://www.unimelb.edu.au/legal/website-terms#problems.

[48] Youtube Terms of Service. https://www.youtube.com/static?template=terms&gl=ID.

[49] Brito M.J.M., da Silva Caram C., Montenegro L.C., Rezende L.C., Rennó H.M.S., Ramos F.R.S. (2017).

[50] Patterson K.D., Pyle G.F. (1991). The geography and mortality of the 1918 influenza pandemic. Bull. Hist. Med..

[51] Martini M., Gazzaniga V., Bragazzi N.L., Barberis I. (2019). The Spanish Influenza Pandemic: a lesson from history 100 years after 1918. J. Prev. Med. Hygiene.

[52] Zylberman P., Phillips H., Killingray D. (2003). The Spanish Influenza Pandemic of 1918–1919: New Perspectives.

[53] Van der Eng P. (2023). Mortality from the influenza pandemic of 1918–19 in Indonesia. https://acde.crawford.anu.edu.au/acde-research/working-papers-trade-and-development.

[54] Tsoucalas G., Kousoulis A., Sgantzos M.N., Kousoulis A., Sgantzos M. (2016). The 1918 Spanish flu pandemic, the origins of the H1N1-virus strain, a glance in history. Euro. J. Clin. Biomed. Sci..

[55] Chandra S. (2013). Mortality from the influenza pandemic of 1918–19 in Indonesia. Popul. Stud..

[56] Lie R. (2022). Surviving the influenza the use of traditional medicines to combat the Spanish flu in colonial Indonesia, 1918-1919. Wacana.

[57] Brown C. (2003).

[58] Barry J.M. (2004).

[59] Tarling N. (2008).

[60] Asmara S.A. (2022). Flu Spanyol Di Jawa 1918-1920: Dari Penyebab, Hoax, Influenza Ordonantie, Hingga Kearifan Lokal Masyarakat Jawa. Mozaik.

[61] Sharifi A., Khavarian-Garmsir A.R. (2020). The COVID-19 pandemic: impacts on cities and major lessons for urban planning, design, and management. Sci. Total Environ..

[62] Harini S., Paskarina C., Rachman J.B., Widianingsih I. (2022). Jogo Tonggo and pager Mangkok: synergy of government and public participation in the face of COVID-19. J. Int. Womens Stud..

[63] Hays J.N. (2005).

[64] Pamungkas F.M. (2021). Satgas Flu Spanyol di Hindia Belanda. Historia.

[65] de Zwart P. (2022).

[66] Gallardo-Albarrán D., de Zwart P. (2022). The regional impact of an epidemic: socioeconomic and demographic data in Java, 1905-1924. Data Brief.

[67] Gallardo-Albarrán D., de Zwart P. (2021). A bitter epidemic: the impact of the 1918 influenza on sugar production in Java. Econ. Hum. Biol..

[68] Brata A.G., Triandaru S., Patnasari Y., Setyastuti R., Sutarta A.E., Sukamto A. (2022). The Spanish flu pandemic and income distribution in Java: lessons from the 1920s. J. Ekon. Malaysia.

[69] Setiawati N.A. (2022). https://www.youtube.com/watch?v=b4TD1y9Ni3E.

[70] Suditomo A. (2021). https://www.youtube.com/watch?v=MhAU2zLgCR4&t=10s.

[71] Setyowati Y.I. (2018). Penyakit Kolera dan Pemberantasannya Di Surabaya Tahun 1918-1942. Ilmu Sejarah.

[72] Hera F.D. (2024). https://nationalgeographic.grid.id/read/132174541/pesjati-takdir-balita-penyintas-pagebluk-pes-di-hindia-belanda?page=all.

[73] Ernaka I., Emalia I., Pradjoko D. (2021). Proceedings of the 9th Asbam International Conference (Archeology, History, & Culture In The Nature of Malay).

[74] Fidiyani M. (2013). Pemberantasan Wabah Penyakit Pes di Lingkungan Penduduk Praja Mangkunegaran Tahun 1915-1929. Avatara.

[75] Wardana I.G.W.W. (2016). Kebijakan Pemerintah Kolonial Dalam Penanganan Penyakit Cacar Di Jawa Abad XIX-XX. Social Studies. Soc. Stud..

[76] Cipta S.E. (2020). Penanganan Pemerintah Hinda Belanda Dalam Menghadapi Berbagai Wabah Penyakit di Jawa 1911-1943. J. Candrasangkala.

[77] Cipta S.E. (2020). Upaya penanganan pemerintah Hindia Belanda dalam menghadapi berbagai wabah penyakit di Jawa 1911–1943. Equilibrium.

[78] Nurwati S.A.H. (2021). Penyakit Tuberkulosis di Surabaya Tahun 1937-1942. Hist. Santiago.

[79] World Health Organization (2021).

[80] Direktorat Jenderal P.P., PL Departemen Kesehatan RI (2007).

[81] Lie R. (2020).

[82] Trein P., Meyer I., Maggetti M. (2019). The integration and coordination of public policies: a systematic comparative review. J. Comp. Policy Anal..

[83] Candel J.J.L., Biesbroek R. (2016). Toward a processual understanding of policy integration. Policy. Sci..

[84] Oktaria V. (2022). Timeliness of routine childhood vaccinations in Indonesian infants in the first year of life. Vaccine.

[85] Admiraal H., Cornaro A. (2020). Future cities, resilient cities – the role of underground space in achieving urban resilience. Underground Space.

[86] Hamidi S., Sabouri S., Ewing R. (2020). Does density aggravate the COVID-19 pandemic?: early findings and lessons for planners. J. Am. Plan. Assoc..

[87] Kraemer M.U.G. (2020). The effect of human mobility and control measures on the COVID-19 epidemic in China. Science.

[88] Suwarno Y., Rahayu N.S. (2021). Is policy integration real in policy practice? Critical review on how government of Indonesia respond to Covid-19 pandemic. IOP Conf. Ser. Earth Environ. Sci..

[89] Djalante R. (2020). Review and analysis of current responses to COVID-19 in Indonesia: period of January to march 2020. Progr. Disas. Sci..

[90] Candel J.J.L. (2017). Holy grail or inflated expectations? The success and failure of integrated policy strategies. Policy Stud.

[91] Cejudo G.M., Trein P. (2023). Pathways to policy integration: a subsystem approach. Policy. Sci..

[92] Biesbroek R. (2021). Policy integration and climate change adaptation. Curr. Opin. Environ. Sustain..

[93] Kementrian PPN & Bappenas (2022).

[94] Ishimaru T. (2022). The impact of COVID-19 outbreak on health emergency and disaster in Japan. Sustainability.

[95] Traore T. (2023). How prepared is the world? Identifying weaknesses in existing assessment frameworks for global health security through a one health approach. Lancet.

[96] Gupta V. (2018). Analysis of results from the joint external evaluation: examining its strength and assessing for trends among participating countries. J. Glob. Health.

[97] Marcotty T. (2013). Intersectoral collaboration between the medical and veterinary professions in low-resource societies: the role of research and training institutions. Comp. Immunol. Microbiol. Infect. Dis..

[98] World Health Organization (2017).

[99] Sironi V.A., Inglese S., Lavazza A. (2022). The ‘one health’ approach in the face of Covid-19: how radical should it be?. Philos Ethics Humanit Med.

[100] Little A. (2012). One health: from theory to practice. Can. Vet. J..

[101] Adnyana I.M.D.M., Utomo B., Eljatin D.S., Sudaryati N.L.G. (2023). One Health approach and zoonotic diseases in Indonesia: Urgency of implementation and challenges. Narra J..

[102] Negara K.S.P. (2022). One health strategy in prevention and control of parasitic zoonosis globally and Indonesia-from theory to practice: a mini-review. Sanglah Gen. Hosp..

[103] Thursina T., Hasanbasri M., Mahendradhata Y. (2024). Strengthening one health: lessons learned of rabies response in Indonesia. BIO Web Conf..

[104] Li H. (2021). Wild animal and zoonotic disease risk management and regulation in China: examining gaps and one health opportunities in scope, mandates, and monitoring systems. One Health.

